# The Added Value of Subcutaneous Peripheral Nerve Field Stimulation Combined with SCS, as Salvage Therapy, for Refractory Low Back Pain Component in Persistent Spinal Pain Syndrome Implanted Patients: A Randomized Controlled Study (CUMPNS Study) Based on 3D-Mapping Composite Pain Assessment

**DOI:** 10.3390/jcm10215094

**Published:** 2021-10-29

**Authors:** Philippe Rigoard, Amine Ounajim, Lisa Goudman, Benedicte Bouche, Manuel Roulaud, Philippe Page, Bertille Lorgeoux, Sandrine Baron, Kevin Nivole, Mathilde Many, Nihel Adjali, Elodie Charrier, Delphine Rannou, Laure Poupin, Chantal Wood, Romain David, Dylan Héraud, Maartens Moens, Maxime Billot

**Affiliations:** 1 PRISMATICS Lab (Predictive Research in Spine/Neuromodulation Management and Thoracic Innovation/Cardiac Surgery), Poitiers University Hospital, 86021 Poitiers, France; Amine.OUNAJIM@chu-poitiers.fr (A.O.); dr.bouche@gmail.com (B.B.); Manuel.ROULAUD@chu-poitiers.fr (M.R.); Bertille.LORGEOUX@chu-poitiers.fr (B.L.); sandrine.baron@chu-poitiers.fr (S.B.); Kevin.NIVOLE@chu-poitiers.fr (K.N.); mathilde.many@chu-poitiers.fr (M.M.); Nihel.adjali@chu-poitiers.fr (N.A.); chantalwood@orange.fr (C.W.); romain-david@hotmail.fr (R.D.); dylan.heraud@etu.univ-poitiers.fr (D.H.); 2 Department of Spine Surgery & Neuromodulation, Poitiers University Hospital, 86021 Poitiers, France; 3 Pprime Institute UPR 3346, CNRS, ISAE-ENSMA, University of Poitiers, 86360 Chasseneuil-du-Poitou, France; 4 Department of Neurosurgery, Universitair Ziekenhuis Brussel, 1090 Brussels, Belgium; lisa.goudman@gmail.com (L.G.); mtmoens@gmail.com (M.M.); 5 STIMULUS Research Group, Vrije Universiteit Brussel, 1090 Brussels, Belgium; Philippe.PAGE@chu-poitiers.fr; 6 Pain Evaluation and Treatment Centre, Poitiers University Hospital, 86021 Poitiers, France; Elodie.CHARRIER@chu-poitiers.fr (E.C.); delphine.rannou@chu-poitiers.fr (D.R.); laure.poupin@chu-poitiers.fr (L.P.); 7 Physical and Rehabilitation Medicine Unit, Poitiers University Hospital, University of Poitiers, 86021 Poitiers, France

**Keywords:** Spinal Cord Stimulation, SubQ-stimulation, Hybrid Stimulation, Multidimensional Pain assessment, Pain Mapping, Failed Back Surgery Syndrome (FBSS), Failed Spinal Cord Stimulation Syndrome (FSCSS), salvage therapy

## Abstract

While Spinal Cord Stimulation (SCS) provides satisfaction to almost 2/3 of Persistent Spinal Pain Syndrome-Type 2 (PSPS-T2) patients implanted for refractory chronic back and/or leg pain, when not adequately addressed the back pain component, leaves patients in a therapeutic cul-de-sac. Peripheral Nerve field Stimulation (PNfS) has shown interesting results addressing back pain in the same population. Far from placing these two techniques in opposition, we suggest that these approaches could be combined to better treat PSPS-T2 patients. We designed a RCT (CUMPNS), with a 12-month follow-up, to assess the potential added value of PNfS, as a salvage therapy, in PSPS-T2 patients experiencing a “Failed SCS Syndrome” in the back pain component. Fourteen patients were included in this study and randomized into 2 groups (“SCS + PNfS” group/*n* = 6 vs. “SCS only” group/*n* = 8). The primary objective of the study was to compare the percentage of back pain surface decrease after 3 months, using a computerized interface to obtain quantitative pain mappings, combined with multi-dimensional SCS outcomes. Back pain surface decreased significantly greater for the ”SCS + PNfS” group (80.2% ± 21.3%) compared to the “SCS only” group (13.2% ± 94.8%) (*p* = 0.012), highlighting the clinical interest of SCS + PNfS, in cases where SCS fails to address back pain.

## 1. Introduction

Spinal Cord Stimulation (SCS) has been used for more than 50 years and is nowadays considered as an effective therapy to treat refractory chronic back and/or leg pain [1,2,3,4,5,6] in Failed Back Surgery Syndrome (FBSS) [7,8,9] or Persistent Spinal Pain Syndrome type 2 (PSPS-T2) patients [10]. While SCS provides satisfaction for 53–62% of implanted PSPS-T2 patients, with 24-month follow-up [11,12], back pain component has always been considered as a variable limitation to SCS efficacy and, when not adequately addressed, leaves patients in a therapeutic cul-de-sac.

Three different scenarios can lead to what could be described as “Failed SCS Syndrome”, with persistent back pain component: (i) First scenario, despite SCS implantation, the patient has never experienced any substantial relief of his back pain; (ii) Second scenario, the patient was initially considered as a good SCS responder since his neuropathic leg component was addressed by neurostimulation and he was not suffering from any kind of backpain at the time of implantation, but he later developed a new pain onset at the back, which became progressively refractory to pain medical management and with no mechanical cause accessible to any etiological treatment, including further spine surgery. (iii) Third scenario, the patient has been previously implanted for back and leg pain, and was initially considered as a positive responder to SCS, but presented a loss of efficacy over time regarding the back pain component, a phenomenon which is often called ‘SCS tolerance’, and reported in up to 20–40% of patients [13].

In this specific context, we can assume that Peripheral Nerve field Stimulation (PNfS) could be considered as salvage therapy for these patients. Indeed, Deer et al. [14] reported in a systematic review of 14 Randomized Controlled Trials (RCTs) that the PNfS approach is safe and relatively effective to treat refractory migraine, cluster headache, shoulder pain, pelvic pain, neuropathic pain of other origins and low back pain. More specifically, McRoberts et al. [15] showed that PNfS could relieve at least 50% of pain intensity for 69.5% of patients (16 out of 23) suffering from chronic intractable back pain at 1 year. More recently, Eldabe et al. [16] documented a responder rate of 56.7% of PSPS-T2 patients after a 9-month period using PNfS added to Optimized Medical Management (OMM). Although these findings clearly show that PNfS can be an interesting option to treat PSPS-T2 patients, with a responder rate relatively similar to SCS, it remains difficult to determine which subgroup of PSPS-T2 patients should benefit the most from one approach or the other, according to practices and reimbursement systems in a given country.

Far from opposing these two techniques to one another, it has been suggested that for a given patient, these approaches could be combined to treat PSPS-T2 and even that a potential synergistic effect of SCS and PNfS could ensue. In this perspective, a prospective multicenter RCT [17] assessed pain intensity decrease in chronic low-back pain patients, after a 6-month period of either PNfS alone (*n* = 28) or a combination of PNfS+SCS (*n* = 74). The authors reported that without any statistical difference PNfS was as effective as the combination of PNfS+SCS to improve refractory pain relief. In another RCT, van Gorp et al. [18] compared SCS with SCS + PNfS in PSPS-T2 patients, for whom a combination of SCS + PNfS techniques was proposed after lead trial, if initial implanted SCS was effective for leg pain (≥50%), but not sufficiently effective for back pain (<50%). Patients implanted with SCS + PNfS reported a higher percentage of responders (42.9%) compared to patients with SCS alone (4.2%), after 3 months of follow-up. All in all, this study showed that PNfS could be used, as an added therapy to SCS, to address a persistent significant low back pain component in PSPS-T2 patients, in whom SCS alone help to reduce leg pain but remains of limited efficacy for the back pain component. However, these encouraging first results deserve further investigation, as patient follow-up in this study was limited to 3 months, and as adding PNfS was proposed starting with initial lead trial, which corresponds to a scenario different from the “Failed SCS Syndrome” we are describing here.

We hypothesized that considering PNfS as a salvage therapy, rather than any another alternative approach or other SCS stimulation paradigm, could offer new opportunities to optimally treat patients already implanted with SCS and suffering from long-term back pain. To validate this hypothesis, we designed a prospective, comparative, randomized controlled study aimed at evaluating the potential added value of PNfS as a means of relieving back pain in PSPS-T2 patients suffering from persistent low back pain despite previous SCS implantation and initial success.

## 2. Materials and Methods

### 2.1. Study Design

CUMPNS is a prospective, randomized controlled open-label monocentric study with a 12-month follow-up period, which was designed to assess the added value of implanting PNfS in PSPS-T2 patients previously implanted with SCS, who did not achieve adequate back pain relief after attempting to achieve optimal pain coverage and relief using all possible SCS programming modalities and optimized medical management.

Patients were enrolled from the University Hospital of Poitiers between February 2013 and April 2017. Informed consent was obtained from all patients before any data collection. The study was conducted in accordance with the Declaration of Helsinki and complied with CONSORT guidelines for RCT, Good Clinical Practice guidelines and was approved by an ethics committee (CPP-Ouest-III and ANSM: 2012-A00532-41). The study is registered at clinicaltrials.gov (NCT02110888).

### 2.2. Patient Selection

#### 2.2.1. Inclusion Criteria

Patients aged between 18 and 80 years were eligible when they were diagnosed as PSPS-T2 (defined as persistent back and leg pain, present for at least 6 months, following one spinal surgical procedure) [19] and for whom OMM (including interventional pain procedures) failed to relieve pain. Patients had to be previously implanted with SCS for chronic neuropathic refractory pain, with a positive response to DN4 questionnaire (DN4 [20], and to have experienced effective pain relief of the leg pain component (≥50%) with SCS, but were still experiencing refractory back pain scored ≥ 40 mm on the Visual Analog Scale (VAS). Several SCS reprogramming sessions had to be performed to attempt targeting the back pain component with SCS (without any loss of efficacy in leg pain), before proposing this study to the patient.

After approving the informed consent, the patient was given the study notebook in order to report baseline characteristics and questionnaires.

#### 2.2.2. Exclusion Criteria

Patients for whom back pain could be treated surgically (discogenic lumbar pain, spinal instability, spinal deformation, etc.) and patients presenting surgical, psychiatric, or anesthetic contraindication to be implanted with a PNfS device were not included. In line with our daily practice, patients who did not receive any Transcutaneous Electrical Nerve Stimulation (TENS) treatment within 6 months and who did not respond to this therapy were likewise excluded. Patients who presented progressive psychosis or history of severe psychosis that required hospitalization, active cancer pathologies, and women of childbearing age without effective contraception (hormonal/mechanical: oral, injectable, transcutaneous, implantable, intrauterine device, or surgical: tubal ligation, hysterectomy, total ovariectomy) were not included either.

### 2.3. Procedures and Additional PNfS Implantation

**Figure 1:**
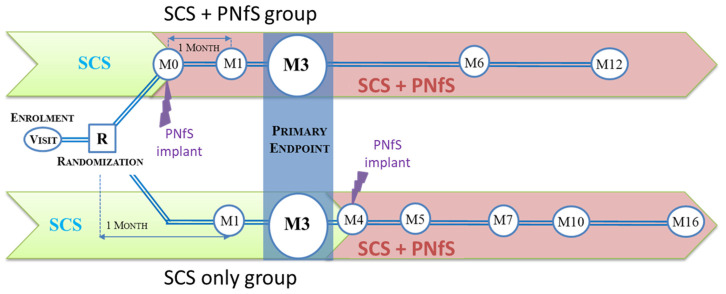
Study design. SCS: Spinal Cord Stimulation; PNfS: Peripheral Nerve field Stimulation.

Eligible patients were randomly assigned to one of two parallel groups in a 1:1 ratio. Randomization was conducted through a clinical data collection website (https://www.dirc-hugo-online.org/csonline, accessed on 31 August 2021) designed for the purpose of this trial, accessible to the investigators through a personal identifier and password. Neither the study medical staff, nor the patients were blinded to the randomization. In addition to SCS device implantation, patients in the intervention group (“SCS + PNfS”) were implanted with PNfS at baseline, whereas patients in the control group (“SCS only”) were implanted with SCS alone for 4 months and then implanted with a PNfS device at the 4-month visit (Figure 1).

**Figure 2:**
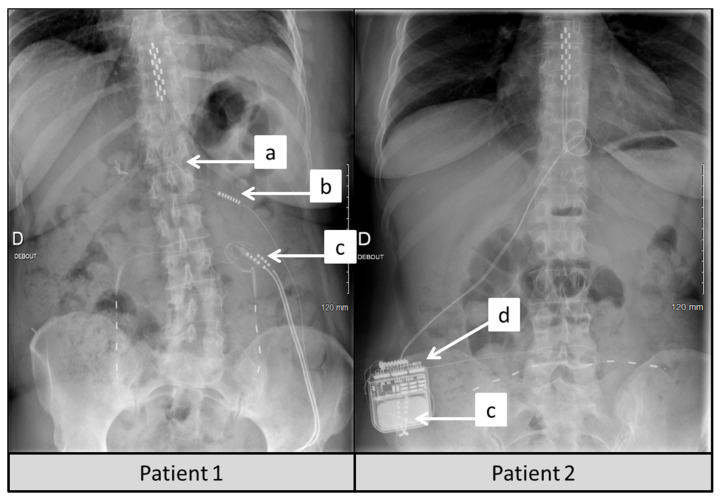
Post-operative X-ray showing technical considerations of SCS + PNfS combination. (**a**) For this first patient, the first channel of the surgical lead wire was disconnected at the lead-extension junction, after selecting the “best” channel to maintain adequate leg pain coverage by reprograming, between the two. (**b**) The second channel, selected as “the best channel” remained connected to the IPG via port *n*2. (**c**) A “Y” extension was implanted to connect the PNfS leads, which were implanted subcutaneously (Quad Plus 4-contact leads, Medtronic) to the 8-contact port *n*1 of the IPG. (**d**) Similarly to Patient 1, the IPG (Restore Advanced, Medtronic) delivers electricity through one channel dedicated to SCS and one other channel dedicated to PNfS for Patient 2.

PNfS implantation consisted in implanting patients with one Octad^®^ lead or two Quad^®^ subcutaneous peripheral stimulation leads (Medtronic, Inc., Minneapolis, MN, USA), depending on the extent of back pain surface. Lead(s) was/were implanted surgically under general anesthesia in prone position. Lead(s) was/were positioned subcutaneously in accordance with back pain surface, documented by tactile informatics pain mapping interface the day before surgery, and was/were connected to an 8-contact extension, which was plugged on the preexisting internal pulse generator (IPG) used for SCS. Since available IPGs contained only two ports at the time of the study, in case the 16 channels (i.e., the 2 ports) were previously used by SCS (two examples of a 16-contact surgical SCS paddle lead are presented in Figure 2), one of the two ports (i.e., 8 channels/16) was carefully selected by ultimate SCS reprogramming and was kept “busy” for continued use of the pre-existing SCS lead in place, the main goal being to preserve optimal leg pain coverage; while the remaining 8 channels were freed in order to be able to connect, on the corresponding port, the 8 plots of the subcutaneous lead(s) to the IPG (Figure 2). Lead programming was repeated at each visit in order to optimize both pain coverage and pain relief for the patients, according to the usual follow-up procedures of patients in our department implanted with a neurostimulation system. Lead programming was conducted in accordance with each patient’s preference. Frequency of stimulation ranged from 40 Hz to 90 Hz, pulse width ranged from 210 μs to 450 μs and stimulation intensity ranged from 0.1 V to 10.5 V.

The “SCS + PNfS” group was followed up at 1, 3, 6, and 12 months. The “SCS only” group was followed up at 1 and 3 months, and after PNfS implantation at 1, 3, 6 and 12 months corresponding to 5, 7, 10 and 16 months from baseline. The two groups were compared at the 1- and 3-month follow-up periods. After the 3-month visit, since all patients were equipped with PNfS implant, pairwise comparisons were performed to assess the benefit of PNfS at 6 and 12 months after implantation.

### 2.4. Study Outcomes

The primary objective of the study was to compare the percentage of decrease of back pain surface between baseline and 3 months between the “SCS only” group and “SCS + PNfS” group. For the purpose of the study, PRISMap software, specifically dedicated to assess pain surface changes objectively, was used to obtain quantitative and comparative metrics [21]. This software has been designed and encapsulated into a tactile computerized interface, to assess patient painful area(s) in terms of intensity (mild, moderate, intense, very intense), real surface (available in cm²) and pain typology characterization (nociceptive/neuropathic pain), with objective, quantitative and reproducible measurements. From a clinical and research perspective, this allows robust comparisons between patients, neurostimulation devices, programs and waveforms, within time, by including a multidimensional composite pain assessment. In this study, pain mapping was collected from a touch screen, where the patient could draw different painful zones, which were then represented as maps and diagrams. The pixels in the patient drawing were then converted into cm², using several anatomical landmarks, patient morphology and morphometry, to measure the pain surface optimally and accurately, using patented data processing system (Patent Applications *n* PCT/EP2014/067231 [22], *n* PCT/FR 14/000 186 [23] and *n* PCT/FR 14/000 187 [24]).

Secondary outcomes, collected at baseline and each visit, included back pain relief assessed with VAS; back pain paresthesia coverage corresponding to the percentage of pain surface covered (or not) by stimulation-generated paresthesia (using N-3D-L^TM^ software/Please see previous publications mentioned above for further details); functional capacity measured with the Oswestry Disability Index (ODI) score [25]; health-related quality of life assessed with the EuroQuol-5Dimensions 3-level (EQ-5D-3L) index [26]; psychological state measured by the Hospital Anxiety and Depression Scales (HADS) [27]; medication intake measured by the third version of the Medication Quantification Scale (MQS-III) [28].

### 2.5. Statistical Analysis

#### 2.5.1. Sample Size

Based on our clinical experience and available data on PNfS use for back pain, we hypothesized that we could expect a difference of 40% between the groups, with a common standard deviation of 25%. With a power of 95% and a significance level of 5%, 19 patients would allow us to detect statistical significance between the groups. Because of the relatively limited follow-up duration before collection of the primary endpoint (3 months), no dropout was considered. Per following these calculations, the original plan was to include 20 patients (10 patients per group).

#### 2.5.2. Statistical Methods

Quantitative variables were described by their means and standard deviations (SD) or by their medians and interquartile range (IQR) depending on the skewness of their distribution. Qualitative variables were described by the number of subjects for each class and their percentage. Normality of all the data was verified using a Shapiro-Wilk test.

The primary endpoint (percentage decrease of back pain surface from baseline to 3 months between groups) was compared using the non-parametric Mann-Whitney test. The secondary quantitative endpoints were compared between the two groups at 1-month and 3-month follow-up using the parametric Student test for low back pain surface, back pain VAS, variation in back pain coverage, ODI score, and HAD score, and the non-parametric Mann-Whitney test for the EQ-5D-3L index.

Qualitative variables were compared between the groups using the Fisher’s exact test.

Quantitative data evaluating the benefit of PNfS at 6 and 12 months after implantation were compared between baseline (inclusion visit for ”SCS + PNfS” group and 3-month visit for “SCS only” group) using a one-factor repeated measures ANOVA or the non-parametric Friedman ANOVA in case of non-normality. When the ANOVA yielded statistically significant results, pairwise comparisons between baseline and 6-month follow-up and 12-month follow-up were conducted using a paired Student test or Wilcoxon test in case of non-normality of the difference between visits. Qualitative variables were compared between visits using a McNemar test.

In the safety analysis, rates of adverse events and severe adverse events were reported. 

A *p*-value < 0.05 was considered as statistically significant. All tests were two-tailed. The analysis was conducted based on an ITT principle.

Statistical analyses were conducted using the R software (Version 3.6.1, R Foundation for Statistical Computing, Vienna, Austria).

## 3. Results

**Figure 3:**
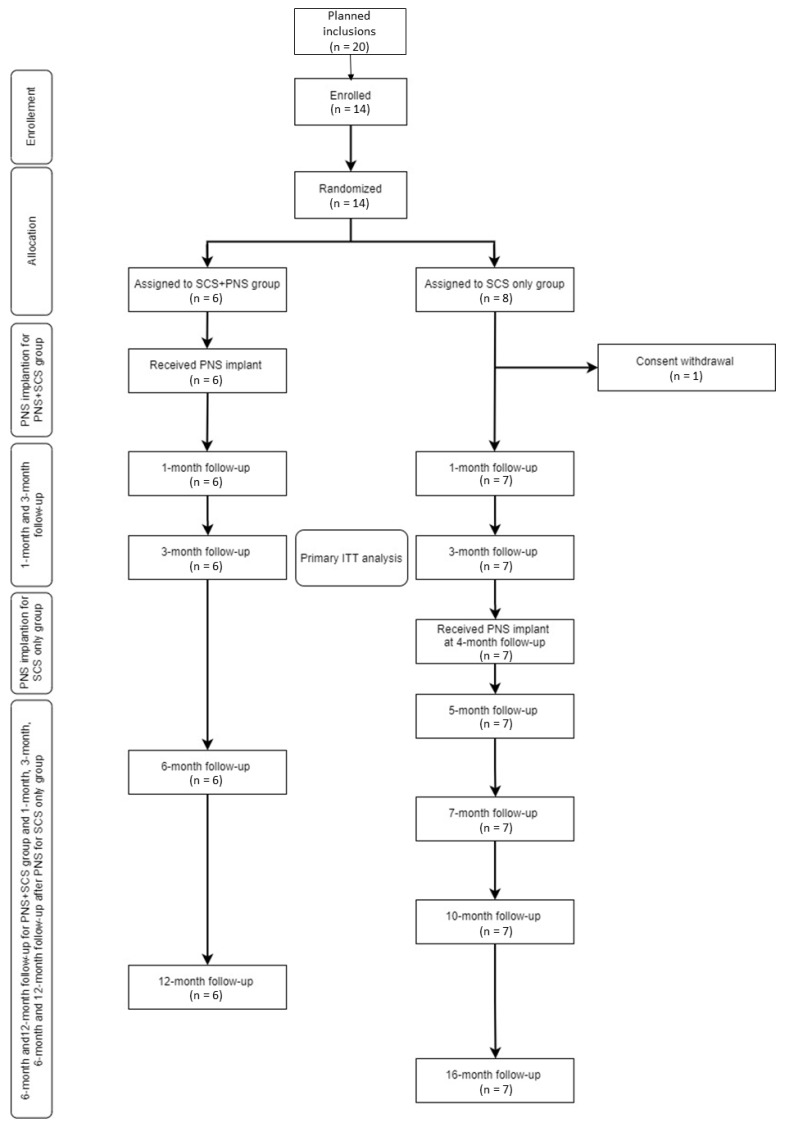
Study Flow chart. SCS: Spinal Cord Stimulation; PNfS: Peripheral Nerve field Stimulation; ITT: Intention-To-Treat.

As developed in the statistical plan, 20 patients were planned to be included in the study initially, but due to prolonged recruitment challenges, we decided to terminate the study after having included 14 patients with complete follow-up. Early study termination was approved by the RCT Steering Committee. Figure 3 presents the flow chart of the patients included in CUMPNS study. The 14 patients included in the study were randomized (*n* = 6 in the “SCS + PNfS” group vs. *n* = 8 in the “SCS only” group). One patient withdrew his consent because he was not compliant with the randomization result. The final analyzed sample consisted in 6 patients in the SCS + PNfS group and 7 patients in the “SCS only” group. No patient was lost during the follow-up period. Baseline characteristics of the patients are presented in Table 1.

### 3.1. Primary Endpoint

The percentage of back pain surface decreased significantly more in the ”SCS + PNfS” group compared to the “SCS only” group at 3 months (*p* = 0.012) (Table 2).

### 3.2. Secondary Endpoints

#### 3.2.1. Between-Group Analyses

The relative and absolute change in secondary and primary outcomes at 1-month and 3-month follow-up for each group are presented in Table 2.

At 1-month, decrease was significantly greater in the “PNfS+SCS” than the “SCS only” for the back pain surface (*p* = 0.003), back pain paresthesia coverage (*p* = 0.017), back pain VAS (*p* = 0.001), ODI score (*p* = 0.034), but not for EQ-5D-3L (*p* = 0.40), HADS anxiety (*p* = 0.190) and depression (*p* = 0.40) scores, or MQS-III score (*p* = 0.07).

Pain VAS at 3-month follow-up was significantly greater in the “SCS + PNfS” compared to the “SCS only” group (*p* <0.001). No difference was observed in the percentage of decrease of leg pain VAS at 3-month between groups (*p* = 0.4). At 3-months, back pain paresthesia coverage increase was significantly greater in the “SCS + PNfS” compared to the “SCS only” (*p* = 0.016). At-3 months, our results did not show any significant difference between “SCS + PNfS” and “SCS only” for the ODI (*p* = 0.07), EQ-5D-3L (*p* = 0.18), HADS depression (*p* = 0.8) and anxiety (*p* = 0.7), or MQS-III score (*p* = 0.07).

#### 3.2.2. Within-Group Analyses

**Figure 4:**
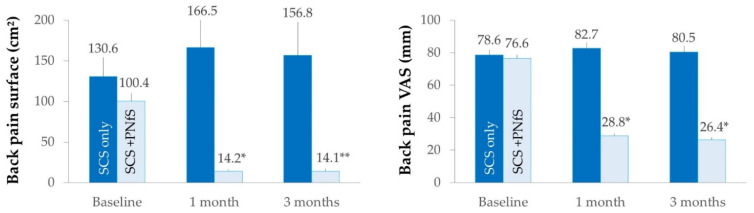
Mean back pain surface in cm² (left panel) and back pain VAS in mm (right panel) and their standard errors at baseline, and at 1- and 3-month follow-up for “SCS only” and “SCS + PNfS” groups. * *p* <0.05; ** *p* <0.01: significant changes compared with baseline. SCS: Spinal Cord Stimulation; PNfS: Peripheral Nerve field Stimulation.

The mean back pain surface and back pain VAS at baseline and at 1 and 3-month follow-up for the two groups are presented in Figure 4. The secondary outcomes at baseline, 1-month and 3-month follow-up are presented in Table 3. 

We found significant change between baseline and 1-month follow-up for the “SCS + PNfS” group in back pain surface (*p* = 0.03), back pain VAS (*p* = 0.03) and ODI (*p* = 0.04), but not in EQ-5D-3L (*p* = 0.06), HADS depression score (*p* = 0.39) and HADS anxiety score (*p* = 0.37). At 1-month, no significant changes were found for the “SCS only” group. At 3-month follow-up, we found a significant change for the “SCS + PNfS” group in back pain surface (*p* = 0.01) and back pain VAS (*p* = 0.03) but not in EQ-5D-3L (*p* = 0.17), HADS depression score (*p* = 0.28) and HADS anxiety score (*p* = 0.78). At 3-months, no significant changes were found for the “SCS only” group.

### 3.3. Paired Comparisons of “SCS + PNfS” and “SCS Only” with a 6- and 12-Month Follow-Up

For this analysis, the data of the two groups were pooled. The mean differences and paired comparisons for all outcomes are presented in Table 4. Repeated measures ANOVA analysis showed a main effect of time of PNfS on back pain surface (*p* = 0.015). Paired comparisons showed that back pain surface significantly decreased at 6-month follow-up after PNfS implantation (*p* = 0.013). The significant decrease in back pain surface observed at 6 months did not persist at 12 months (*p* = 0.27). 

Repeated measures ANOVA analysis showed a main effect of time of PNfS on back pain VAS (*p* < 0.0001). In the paired comparisons, we found a significant difference of back pain VAS between baseline and 6 months (*p* = 0.0003) and 12 months (*p* = 0.001) following PNfS implantation. Time effect of PNfS on leg pain VAS score was not significant (*p* = 0.7).

Repeated measures ANOVA analysis showed a main effect of time of PNfS on ODI score (*p* = 0.001). The ODI score showed a significant decrease at 6 months (*p* = 0.02) and 12 months (*p* = 0.03) after PNfS. 

Repeated measures ANOVA analysis showed a main effect of time of PNfS on EQ-5D-3L score score (*p* = 0.03). The EQ-5D-3L score showed a significant increase between baseline and the 6-month (*p* = 0.017) but not between baseline and 12-month (*p* = 0.1) follow-up. Repeated measures ANOVA analysis showed a main effect of time effect of PNfS on HADS anxiety score (*p* = 0.006). Anxiety score showed a significant decrease 6 months (*p* = 0.03) and 12 months (*p* = 0.008) after PNfS implantation from baseline. Time effect of PNfS on HADS depression score was not significant (*p* = 0.5). 

### 3.4. Safety

All in all, 33 adverse events (AE) were reported during the study of which 4 were severe. The most frequent AE was postoperative pain at the site of lead/IPG implantation (15.2%), followed by falling (12.1%) and early depletion of IPG battery (9.1%), fatigue induced by stimulation (6.1%), digestive disorders (6.1%), nausea with headache (3.0%), leakage and delayed healing in the left lumbar scar (3.0%), bursitis of the gluteus medius (3.0%), hematoma in the lumbar region (3.0%), displacement of the neurostimulation device (3.0%) and others AE such as allergy to TENS patch, unstable diabetes, anxiety, etc. (36.4%). No infection occurred and no patient required any explantation of the PNfS or SCS device during this study.

## 4. Discussion

Our study shows that PNfS added to SCS can significantly reduce back pain surface by more than 80% in comparison with SCS alone after 3 months in PSPS-T2 patients, already implanted with SCS and experiencing SCS failure to address their back pain component. Long-term follow-up (6 and 12 months) results also showed a significant decrease of pain intensity, a significant increase of back pain paresthesia coverage provided by the combination of PNfS and SCS, and a significant decrease of ODI and Anxiety scores.

### 4.1. Back Pain: A Real Target for Neurostimulation? Episode 2

While SCS has shown some limitations in pain relief specifically for the back pain component in the past [29,30], PNfS has more recently demonstrated an interesting level of evidence to treat refractory back pain effectively [14]. In opposition to SCS, PNfS should be considered as a peripheral neurostimulation technique, since it targets small peripheral nervous branches, distributed randomly at the subcutaneous interface between skin layers (including hypoderma) and musculoskeletal fascia and aponeurosis. This neurostimulation technique indeed targets distal branches of the peripheral nervous system and aims at delivering permanent electrical “field” stimulation directly at the center of maximal pain area via subcutaneously inserted lead(s) [31,32]. In contrast, SCS is considered as a central nervous system approach, requiring access to the spinal canal, to deliver electrical current via electrode(s) placed in the dorsal epidural space [1,4]. The concept of combining peripheral and central stimulation to modulate peripheral pathways and neural structures, at the level of the injury / central pathways and neural structures, above the lesion appears appealing as a synergistic approach. This could represent an alternative offering new perspectives of managing patients suffering from PSPS-T2, especially in case of SCS Failure. This also reactivates a debate regarding the place of the different neurostimulation techniques in our therapeutical armentorium: Beyond these questions: how to select SCS candidates, the type of lead to be implanted, IPG, program(s), waveform(s), and the surgical approach, have we thought about which neural target to stimulate, as a pre-requisite?

From PNfS, through Peripheral Nervous System, Dorsal Root Ganglion, SCS, Deep Brain Stimulation and Motor Cortex Stimulation, it appears that the selection of the appropriate target, all along the pain nociceptive transmission pathway, becomes more and more difficult as time goes by. Given this nebulosity, it would be artificial but useful to distinguish: (a) a central SCS approach, mainly targeting neuropathic pain components, affecting the periphery (such as the leg pain component, resulting directly from a clearly identified L4, L5 or S1 nerve root lesion, which would expresses its neuropathic component on the corresponding dermatomal distribution of its anterior branches, becoming lombo-sacral or cervical plexus afferences) from (b) a peripheral PNS/PNfS approach, mainly targeting mixed nociceptive and neuropathic pain components, affecting axial dermatomal distribution of the trunk (such as the back component in PSPS-T2 patients), which is characterized by a pain typology, which is rarely pure, often mixing neuropathic and mechanical features, insofar as this anatomical organization depends on axial architecture, centered on spine anatomy (a complex musculo-skeletal structure), and for which innervation depends on the posterior branches of the corresponding nerve roots, in opposition to (a) [9,33,34,35]. This complex puzzle might require several conceptual approaches, dedicated to several anatomical components with a distinct innervation, defining “neuro-compartments”, so as to involve separate targets of neurostimulation, requiring different mechanisms of action and combinations. However, the lack of possible comparisons between the different techniques, on the same implanted patient, euphemizes the potential impact of practical considerations and reinforces the need for RCTs, involving several targets, addressed by several techniques, eventually combined, on the same individual [36]. That is precisely the purpose of such a study.

### 4.2. Mechanical and Neuropathic Back Pain Component Typology Patient Characterization Suggests a Specific Role of PNfS on Mechanical Back Pain Features, as a Synergistic Approach

It is well-established that radicular pain refers mainly to neuropathic pain, whereas the nature of back pain can be associated with neuropathic and/or mechanical pain. Our results suggest that the neuropathic pain component corresponding to the leg level could be adequately treated by SCS and it appeared legitimate, as SCS was failing to relieve pain in these PSPS patients, to try using PNfS for neuropathic/mechanical residual pain components localized at their back. The potential overlap between SCS and PNfS efficacy on the same patient has yet to be the subject of analysis and publication. 

In this context, we observed that patients experienced a substantial gain in function after PNfS implantation, associated with back pain coverage increase, back pain decrease and improvement in quality of life. In parallel, it is interesting to note that whereas all patients had a positive global DN4 neuropathic assessment at the time of SCS implantation and during their follow-up, only 8/13 patients had a specific back pain DN4 positive score, before additional PNfS implantation. Similar back pain relief was observed between patients with positive and negative back pain DN4, that clearly shows that SCS + PNfS appears to work not only on back pain neuropathic features, but also on mechanical aspects. The differential response on this potential partially mechanical back pain component is a strong argument for potential synergistic action of SCS + PNfS combination, especially when significant residual mechanical back pain occurs, and also a strong argument confirming that SCS alone is not working well on mechanical aspects (−13.2% of back pain decrease for SCS alone vs. 80.2% for SCS + PNfS), as largely suggested in the literature [29,30].

We would carefully hypothesize that PNfS, as peripheral nerve stimulation, implanted in front of paravertebral muscles, innervated fascia and aponeurosis, could have an electrical influence on proximal structures, and could play an indirect role as a spinal musculoskeletal system electronic booster, helping to regain function by improving muscular adaptation and to increase proprioceptive feedback coming from this complex musculature. This concept has been the subject of new developments in peripheral nerve stimulation, as published by Eldabe’s team [37,38], and could be transposed to the differential role and added-value of PNfS to SCS. 

We could also hypothesize that PNfS could retrogradely influence other spinal structures, likewise innervated by the posterior distribution of the spinal nerves, which constitute the “electrical vehicle” transporting PNfS information towards the central nervous system, up to DRG and the Spinal Cord (SC) junction [34,35]. These anatomical structures correspond to the facet joints (innervated by posterior articular ramii), the dural envelopes and the corresponding intervertebral discs (innervated by Luschka’s nerve), as described in an anatomical review of the structures potentially involved in post-operative back pain [35]. Lastly, we could discuss the notion of temporality, leading to chronification of pain, not regarding the biopsychosocial dimension but rather the neural circuitry plasticity induced by the inaugural nervous lesion, which defines neuropathic pain genesis [39] and leads to progressive chemical and structural changes over time. This concept supports the notion of primary and secondary hyperalgesia [9], predisposing to future allodynia at a later stage in the temporal process, and it could be transposed to this study. In a temporal sequence and as a stable lesioned component, SCS would have been an effective tool to address the neuropathic leg pain component, whereas back pain would still remain under “transformation” from a mechanical predominant component to the progressive development of the above-described neuropathic chemical and then structural plasticity, thereby explaining the limited response to SCS, since central neural plasticity has yet to appear, and also explaining the better response to PNS, since peripheral plasticity precedes central plasticity [9]. While the above extrapolations would need more robust substrate to be documented, they could eventually influence our choice of neural structures to target and, consequently, our choice of neurostimulation technique in favor of PNS, if and when the mechanical component is still present, if not predominant, on an axial dermatomic painful distribution. Objective pain mapping tools, including pain typology characterization, would be of great help to design future research.

### 4.3. The Predictive Role of TENS before Considering Implanted Neurostimulation, with a Focus on PNfS

Interestingly, echoing on the previous discussion, as a non-invasive tool, the TENS application can be placed on specific residual pain locations, eventually mechanical ones, in patients already implanted with SCS. This is desirable for two main reasons. First, it matters to try to recapture a therapeutical effect when SCS shows its limitations, and as a symptomatic treatment for this population, TENS is altogether non-invasive. However, patients are exposed to long-term skin allergy or discomfort [40,41], decreased efficacy over time [42,43,44,45,46], and a limitation on the functional impact of stimulation insofar as TENS constraints represent a concrete burden, limiting daily life activities. This factor would constitute a major argument in favor of switching to implanted stimulation. Second, regardless of the pathophysiological hypothesis supporting the different mechanisms of action of SCS and PNfS, TENS might be considered as a positive predictive tool before implanting PNfS, as it has been studied for SCS over the last decade [32,47]. Mathew et al. [47], for example, showed that TENS can be used to assess patient ability to tolerate paraesthesia induced by SCS. Furthermore, assuming that SCS, PNfS and TENS have commonly based mechanisms of action, since they relay information along the same nociceptive pathway (a classical example is the gate control theory) [48], it is not unreasonable to assume that a positive TENS trial would lead to optimized PNfS and/or SCS outcomes, especially on a tonic-based argumentation. This explains the choice we made to recruit our patients in this study, after a TENS positive trial on the back.

### 4.4. Technical Considerations to Take into Account, When Converting a Patient Already Implanted with SCS to SCS + PNfS

As illustrated on Figure 2, implanting additional subcutaneous leads to the existing SCS system requires anticipation and careful management. First, the limited amount of available ports and channels on the IPG, might lead the clinical team to reprogram the existing SCS device, and to disconnect one of the two channels dedicated to SCS in order to make one port free for a PNfS extension. Second, the limited capabilities of generating pain surface coverage (having the size of a credit card) implies that the implanter carefully select the “triggers” and most sensitive painful areas to cover with subQ-stim, the objective being not to disappoint the expectations of patients, who would like to observe a pain decrease on the entire back pain surface, which is sometimes disproportionate compared to PNfS possibilities [14,32]. This represents a clear limitation of CUMPNS strategy. Third, due to the relative distance between the subcutaneous implanted lead and neural micro-structures to target, with hazardous distribution in a fatty environment, a considerable loss of energy occurs. This explains why the addition of PNfS would require much higher battery capabilities and expose the patient to early battery depletion and a need for replacement. This energetic aspect delineates a clear limitation to the CUMPNS approach, initially designed in 2011, due to the launch of new waveforms, in order to change the paradigm of the temporal resolution of the electrical signal delivered to the SC. The new waveforms also require a sizable amount of energy, which constitutes a real limitation if PNfS was added previously. Lastly and but most importantly, any new surgical procedure, any new material implanted can drastically increase complication rate in a vulnerable population of multi-operated patients [49,50]. As an illustration, we reported pain located at the lead and IPG implantation site for 15.2% of the patients and early pacemaker battery failure for 9.1% of the patients in this study. Fortunately, no infection occurred, but at the extreme, in case of infection, the worst scenario could lead to total explantation of SCS + PNfS system, which would leave these patients in a dramatically worsened condition. These parameters must be carefully taken into account (1) to find the best compromise between invasiveness and patient objectives, and (2) to entrust these techniques to experienced physicians in experimented neuromodulation centers.

### 4.5. Study Limitations

Despite its originality and robust methodology, this study presents some limitations.

#### 4.5.1. PNfS and SCS Compatibility

Up until now, only a few devices have allowed physicians to plug subcutaneous leads without substantial impact on SCS programming capacity. Indeed, the vast majority of IPGs comprise 2 ports to welcome 2 channels of stimulation, enabling management of one 8-contact cylindrical SCS lead and one 8-contact PNfS lead or two leads with 4 contacts. But in the event that a patient has already been implanted with a more sophisticated lead, whether a 16-contact cylindrical SCS lead or a surgical paddle lead, any attempt at implanting PNfS weaken the opportunities offered by the SCS device by reducing the number of available contacts to deliver the electrical current, and consequently restricting the number of programming capabilities. As detailed above, we were compelled to disconnect one channel in order to be able to plug in the PNfS devices used by most of our patients. While our results did not show any decrease of SCS efficacy on leg pain component after 12 months, we cannot rule out a potential loss of efficacy over time, which would necessitate finding a new spatial target(s) of stimulation localized under deliberately inactive contact of the initially implanted SCS lead. Long-term follow-up would thereby be necessary to determine the possible loss of efficacy over time. To prevent patient this occurrence, a new generation of IPGs has been specifically developed over recent years, adding two channels to support the use of two 8-contact PNfS leads if required. These IPGs represent a great opportunity to add PNfS without losing what SCS has accomplished for the patient and will avoid research bias in future studies, bias arising from inter-individual outcome variability due to technical limitations and variability.

#### 4.5.2. Methodological Limitations

First, due to paresthesia generated under tonic stimulation, it was impossible to blind the SCS + PNfS combination. This was the price to pay to respect a RCT design with techniques combined on the same patient, in the context of a “salvage therapy” concept. 

Secondly, the patients assigned to the “SCS only” group might be susceptible to developing a waiting effect. However, this limitation could rather be considered as a potential strength of this study since patients were their own control and since it has been demonstrated that waiting effect in chronic pain patients can also impact negatively on patient outcome. In this scenario, our conclusions and the added value of PNfS would be reinforced.

Our last study limitation arises from the sizable length of time since the initial design of this study in 2011, the challenging recruitment of patients between 2013 and 2017, and the confusion which appeared in the implantation community, exactly at the same time of this study. Indeed, after having spent four decades of SCS trying to find the best spatial neural target to stimulate [1,2,51,52,53,54,55], with an emphasis on lead design [53] and electrical field modeling [56,57], around 2014 neuromodulation philosophy adopted a radically different direction, due to innovations in the temporal resolution of the signal, thereafter focusing on new waveforms. Burst stimulation appeared in 2013 [58], paresthesia free-high frequency techniques were developed contemporarily and it took about 5 years for our community to publish counter-studies, counterbalancing the “overly promising” initial results of some of these techniques [59,60,61,62], the objective being to orient us toward the right compromise for our patients, mixing the two approaches as complementary tools: (i) Spatial resolution can be adjusted by the choice of the neural target and new programing capabilities, based on electrical fragmentation of the current; and (ii) temporal resolution can be adjusted by the IPG, as an alternative to tonic conventional SCS or a combination of several waveforms delivered to the patient, the objective being to enhance our ability to personalize SCS therapies [2,63]. As a consequence, some recently marketed adapters play on the temporal resolution of SCS and give the patient the opportunity to extensively test the different existing waveforms with promising results [64,65,66]. These new insights need to be integrated in our approach to reflect the state of the art, since this paper’s ambition is to propose a salvage algorithm.

### 4.6. Proposal of a Salvage SCS Algorithm for Back Pain Component

As a synthesis of our clinical experience and the research conducted over the last 10 years, we present two different algorithms built at a 10-year interval. 

**Figure 5:**
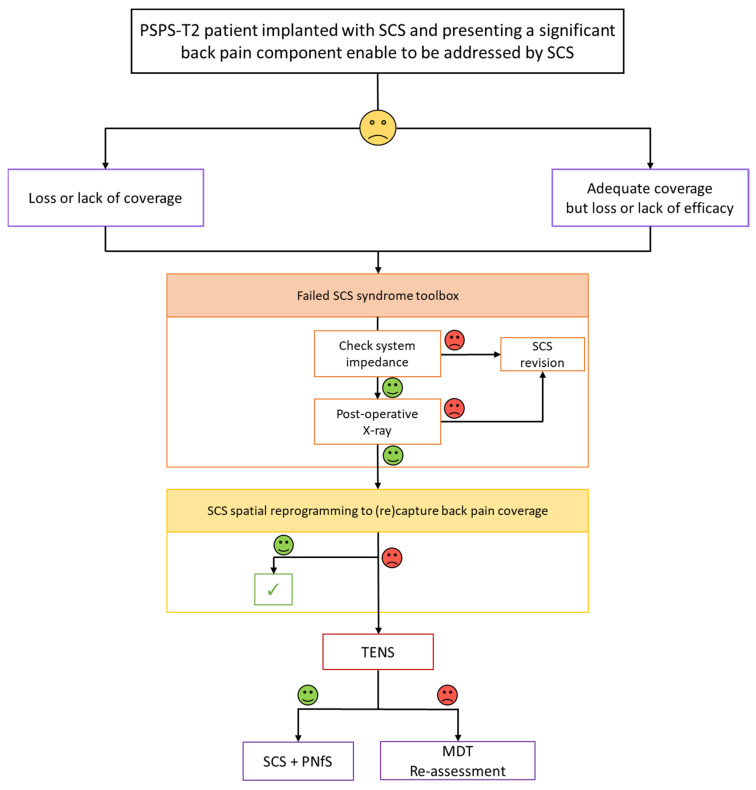
Proposal of a salvage algorithm for persisting back pain despite SCS: 1st version designed in 2011. SCS: Spinal Cord Stimulation; PNfS: Peripheral Nerve field Stimulation; TENS: Transcutaneous Electrical Nerve Stimulation; MDT: MultiDisciplinary Team.

The first algorithm (Figure 5) corresponds to the initial view we had on the notion of salvage therapy in 2011, as we were designing the CUMPNS study. Only tonic conventional stimulation was available at that time. As regards a PSPS-T2 patient, already implanted with SCS, with adequate coverage and pain relief of the leg pain component but insufficient pain coverage and/or pain relief of his back component, our approach consisted in: (1) checking impedance and hardware dysfunction before (2) attempting to reprogram the patient, using a spatial retargeting approach, given the possibilities of the IPG and existing lead and, in case of “Failed SCS Syndrome” (FSCSS), as regards the back component, (3) proposing to add TENS to SCS, as a predictive screening trial in the framework of a potential CUMPNS approach following a positive trial by combining PNfS implantation using the existing SCS system. With this salvage algorithum in mind, we proposed to reassess refractory patients in a MultiDisciplinary Team (MDT) context, thereby reconsidering patient selection and ruling out any etiology that would require an approach different from neurostimulation, in light of re-imaging, clinical evaluation, including a new psychological assessment (Figure 5).

**Figure 6:**
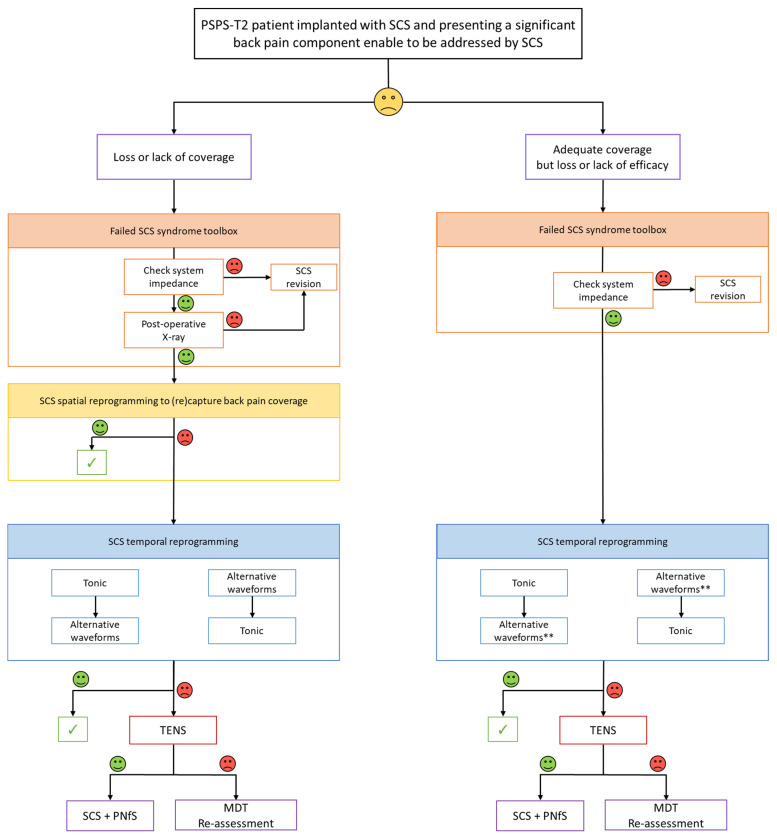
Updated version in 2021, according to technological advances regarding the new waveform paradigm to change the temporal resolution of the electrical signal delivered by the IPG [66]. SCS: Spinal Cord Stimulation; PNfS: Peripheral Nerve field StimulationTENS: Transcutaneous Electrical Nerve Stimulation; MDT: MultiDisciplinary Team.

In 2021, given the new context brought about by recent changes in the paradigm of neurostimulation (i.e., new isolated waveforms, or associated waveforms/Figure 6), our approach regarding salvage options to consider for a FSCSS patient is slightly different. Inspired by the previous algorithm and using a similar substrate, our approach now consists in: (1) checking impedance and hardware dysfunction before (2) attempting to reprogram this patient, using either a spatial and/or a temporal retargeting approach, if the patient is complaining of a loss of coverage, or a temporal resolution retargeting approach, if the patient is complaining of a loss in SCS efficacy, despite adequate back pain coverage, given the possibilities of the IPG and existing lead(s). This approach could require SCS system reexploration and, for some patients, conversion using an implanted adapter, after a new external lead trial [64,65,67,68,69].

It is only in case of “Failed SCS Syndrome” (FSCSS) on the back component, refractory to spatial and temporal retargeting that: (3) we would propose to add TENS to SCS as a predictive screening trial, of our potential CUMPNS approach, using the same modalities as those described above (for Figure 5). All algorithm proposals can be the substrate of discussion but will require further research.

## 5. Conclusions

Adding PNfS to existing SCS in previously successfully implanted PSPS-T2 patients, when back pain component remains difficult or impossible to address despite multiple SCS reprogramming, appears promising as a salvage therapy. In this study, we were able to document patient improvement using objective quantitative measurements, and to correlate 3D back pain surface decrease to patient pain relief using multidimensional composite pain assessment tools. The place of PNfS, in the therapeutical armentorium should be discussed as a potential added-value to existing SCS but might benefit from further clarification, especially given the new temporal modalities, which can now be tried by simple reprogramming and/or connecting to an adapter, depending on implanted devices and patient preference. These various strategies appear as promising as their goal is noble: pushing back the technological limitations to be able to convert SCS failure into new success. However, these new insights need robust studies to document clear added-value and establish legitimacy for the novel modalities.

## Figures and Tables

**Table 1:** Baseline characteristics of the study groups.

Variable at Baseline	SCS + PNfS Group (*n* = 6)	SCS Only Group (*n* = 7)	*p*-Value
Gender			0.56
Male	2 (33.3%)	1 (14.3%)	
Female	4 (66.7%)	6 (85.7%)	
Age (years)	51.7 ± 12.2	45.1 ± 9.5	0.39
Body mass index (kg/cm²)	28.8 ± 6.7	29.0 ± 2.8	0.95
Time between pain onset and SCS implantation (years) *	6 ± 5.3	4 ± 12.5	0.83
Time between SCS and PNfS implantation (years)*	1.5 ± 1.75	2 ± 2	0.38
Back pain DN4 Positive (≥4)	5 (83.3%)	3 (42.9%)	0.27
Back pain DN4 Negative (<4)	1 (16.7%)	4 (57.1%)	
Back pain VAS (/100 mm)	76.6 ± 18.0	78.6 ± 14.6	0.99
Back pain surface (cm²)	100.4 ± 57.6	130.6 ± 162.4	0.84
Baseline back pain paresthesia coverage (%)	0.0 ± 13.2	8.9 ± 12.4	0.21
ODI score (%)	50.3 ± 11.8	48.3 ± 16.1	0.83
EQ-5D-3L index	0.34 ± 0.20	0.46 ± 0.26	0.43
Anxiety HADS score	10.3 ± 3.1	6.3 ± 4.6	0.15
Depression HADS score	8.3 ± 4.1	5.4 ± 2.5	0.25
MQS III score	21.5 ± 14.8	15.8 ± 17.1	0.28
SCS Lead implantation level			0.33
T8	1 (16.7%)	0 (0%)	
T9	3 (50.0%)	5 (71.4%)	
T10	2 (33.3%)	0 (0%)	
T11	0 (0%)	1 (14.3%)	
T12	0 (0%)	1 (14.3%)	

* Median (IQR). DN4: Douleur Neuropathique en 4 questions; EQ-5D-3L: EuroQol 5-Dimensions 3-Level; HADS: Hospital Anxiety and Depression Scale; ODI: Oswestry Disability Index; VAS: Visual Analog Scale; MQS: Medication Quantification Scale.

**Table 2:** Absolute or percentage of decrease for the primary and secondary endpoints comparisons between the “SCS + PNfS” group and “SCS only” group at 1- and 3-month follow-up.

	SCS + PNfS Group (*n* = 6); Mean ± SD	SCS Only Group (*n* = 7); Mean ± SD	*p*-Value
**At 1 month**			
Back pain surface	−89.2 ± 9.4%	−19.3 ± 84.9%	0.003
Back pain paresthesia coverage	10.5 ± 17.0%	−8.9 ± 15.3%	0.017
Back pain VAS	−65.4 ± 20.4%	8.6 ± 26.6%	0.001
ODI score	−33.8 ± 39.9%	12.3 ± 41.1%	0.034
EQ-5D-3L index	0.20 ± 0.30	−0.04 ± 0.27	0.40
HADS anxiety score	0.83 ± 2.66	2.0 ± 1.03	0.190
HADS depression score	1.67 ± 3.67	0.0 ± 1.38	0.40
MQS-III score	−3.57 ± 5.63	3.94 ± 6.73	0.07
**At 3 months**			
Back pain surface	−80.2 ± 21.3%	13.2 ± 94.8%	0.012
Back pain paresthesia coverage	16.05 ± 16.16%	−0.94 ± 2.2%	0.016
Back pain VAS	−68.8 ± 19.9%	4.0 ± 15.0%	<0.0001
ODI score	−31.5 ± 34.1%	5.0 ± 29.7%	0.07
EQ-5D-3L index	0.23 ± 0.33	0.02 ± 0.17	0.18
HADS anxiety score	−0.33 ± 2.34	0.14 ± 2.79	0.70
HADS depression score	1.83 ± 4.07	0.57 ± 1.51	0.80
MQS-III score	−6.32 ± 10.72	7.29 ± 12.20	0.07

EQ-5D-3L: EuroQol 5-Dimensions 5-Level; HADS: Hospital Anxiety and Depression Scale; ODI: Oswestry Disability Index; VAS: Visual Analog Scale; MQS: Medication Quantification Scale.

**Table 3:** Secondary outcomes (mean ± SD) at baseline, 1- and 3-month follow-up for the “SCS + PNfS” and “SCS only” groups.

Endpoints	SCS + PNfS Group (*n* = 6)			SCS Only Group (*n* = 7)		
	Baseline	1-Month	3-Month	Baseline	1-Month	3-Month
Leg pain VAS (mm)	23.5 ± 26.6	19.7 ± 18.7	25.1 ± 25.4	31.5 ± 13.6	33.5 ± 20.1	42.1 ± 22.0
ODI score	50.3 ± 11.8	35.0 ± 21.2 *	37.3 ± 24.0	48.3 ± 16.1	49.1 ± 8.2	46.9 ± 8.6
EQ-5D-3L score	0.34 ± 0.20	0.54 ± 0.24	0.57 ± 0.29	0.46 ± 0.26	0.42 ± 0.21	0.48 ± 0.21
HADS anxiety score	10.3 ± 3.1	9.5 ± 2.9	10.7 ± 4.0	6.3 ± 4.6	4.3 ± 4.2	6.1 ± 4.0
HADS depression score	8.3 ± 4.1	6.7 ± 5.0	6.5 ± 5.5	5.4 ± 2.5	5.4 ± 2.3	4.9 ± 2.9

* *p* <0.05: significant change compared with baseline. VAS: Visual Analogic Scale; ODI: Oswestry Disability Index; EQ-5D-3L: EQ-5D-3L: EuroQol 5-Dimensions 3-Level; HADS: Hospital Anxiety and Depression Scale.

**Table 4:** Paired comparisons of the primary and secondary outcomes between baseline and 6- and 12-month follow-up.

Endpoints	Difference between Baseline and 6-Month Follow-Up			Difference between Baseline and 12-Month Follow-Up		
	Difference	CI95%	*p*-Value	Difference	CI95%	*p*-Value
Back pain surface	−79.81 cm²	(−182.92; 23.30)	0.013	−32.98 cm²	(−107.61; 40.66)	0.27
Back pain VAS	−41.6 mm	(−59.4; −23.8)	0.0003	−39.4 mm	(−57.7; −21.0)	0.001
Leg pain VAS	−5.8 mm	(−21.7; 10.1)	0.4	−2.2 mm	(−18.9; 14.5)	0.8
ODI score	−11.9%	(−21.6; −2.1)	0.02	−10.8%	(−20.6; −1.1)	0.03
EQ-5D-3L score	0.19	(0.04; 0.33)	0.017	0.16	(0.02; 0.34)	0.1
HADS anxiety score	−2.1	(−3.9; −0.3)	0.03	−2.0	(−3.3; −0.7)	0.008
HADS depression score	−0.9	(−3.3; 1.4)	0.4	−1.0	(−3.5; 1.6)	0.4

EQ-5D-3L: EuroQol 5-Dimensions 5-Level; HADS: Hospital Anxiety and Depression Scale; ODI: Oswestry Disability Index; VAS: Visual Analog Scale.

## Data Availability

Not Applicable.
